# Low-grade myofibroblastic sarcoma of the levator scapulae muscle: a case report and literature review

**DOI:** 10.1186/s12891-020-03857-3

**Published:** 2020-12-10

**Authors:** Hirotaka Yonezawa, Norio Yamamoto, Katsuhiro Hayashi, Akihiko Takeuchi, Shinji Miwa, Kentaro Igarashi, Mickhael Bang Langit, Hiroaki Kimura, Shingo Shimozaki, Takashi Kato, Sei Morinaga, Yoshihiro Araki, Yohei Asano, Hiroko Ikeda, Takayuki Nojima, Hiroyuki Tsuchiya

**Affiliations:** 1 Department of Orthopaedic Surgery, Graduate School of Medical Sciences, Kanazawa University, 13-1 Takara-machi, Kanazawa, Ishikawa 920-8640 Japan; 2 Department of Orthopedics, Philippine Orthopedic Center, Quezon City, Manilla Philippines; 3 Department of Diagnostic Pathology, Kanazawa University Hospital, Kanazawa, Japan

**Keywords:** Low-grade myofibroblastic sarcoma, Levator scapulae muscle, Infiltrative pattern, Intramuscular, Wide excision

## Abstract

**Background:**

Low-grade myofibroblastic sarcoma (LGMS) is described as a distinct atypical myofibroblastic tumor often with fibromatosis-like features and predilection for the head and neck, especially the oral cavity and larynx. LGMS arising in the levator scapulae muscle is extremely rare.

**Case presentation:**

A 69-year-old woman was admitted to our hospital because she noticed a hard mass in her left neck six months prior. Magnetic resonance images (MRI) showed a soft tissue tumor of the left levator scapulae muscle. A core needle biopsy showed cellular fascicles or a storiform growth pattern of spindle-shaped tumor cells with minimally atypia. Immunohistochemistry revealed focally positive for α-smooth muscle actin (α-SMA), negative for S-100, and a low-grade spindle cell sarcoma was suspected. Following a biopsy, the tumor was resected with a wide surgical margin. Immunohistochemical staining was a positive for vimentin and α-SMA and negative for desmin, CD34, nuclear β-catenin, and h-caldesmon. LGMS diagnosis was determined based on the histopathological findings. The patient was alive with no evidence of disease eight years after the surgery.

**Conclusions:**

To the best of our knowledge, this is the first case report of LGMS arising in the levator scapulae muscle. In addition to the case report, 48 reports with 103 LGMS cases are reviewed and discussed. In previous reports of LGMS, there were 43 females and 60 males, with a mean age of 43.0 years (range, 2–75). There were 13 (12.6%) patients aged < 18 years, 67 (65.1%) patients aged 18 to 59 years, and 23 (22.3%) patients aged ≥60 years. The average tumor size was 4.4 cm (range: 0.4–22.0). The commonest sites of LGMS was the tongue. Tumor growth patterns were evaluated in 52 cases, and 44 cases (84.6%) showed infiltrative growth patterns. Local recurrence was 26.7%, and distant metastasis was 4.4%. Because of the locally aggressive feature, it is important to diagnose LGMS with biopsy and to excise the tumor with an adequately wide margin.

## Background

Low-grade myofibroblastic sarcoma (LGMS) is a rare and relatively new entity that was recently recognized as such and described by Mentzel et al. [1] in 1998. LGMS is described as a distinct atypical myofibroblastic tumor, often with fibromatosis-like features and predilection for the head and neck [2]. Oral cavity [1, 3–16] and larynx [17–20] cases are common, but a variety of tissues, including skin [21–23], breast [24, 25], vulva [26, 27], parapharyngeal space [28], jaw [1, 10], nasal cavity [29], paranasal sinus [29], soft tissue of the cheek [7, 23], and palate [3, 30] have also been reported. LGMS has been characterized as having a low-grade malignant potential, the propensity to recur locally, and a low likelihood of distant metastases [1, 6, 31]. Treatment primarily involves surgical resection with clear margins [32]. We encountered a case of LGMS of the levator scapulae muscle. To the best of our knowledge, this is the first case report of LGMS arising in the levator scapulae muscle. The present study details the case of a patient with LGMS and reviews 103 relevant LGMS cases.

## Case presentation

**Fig. 1:**
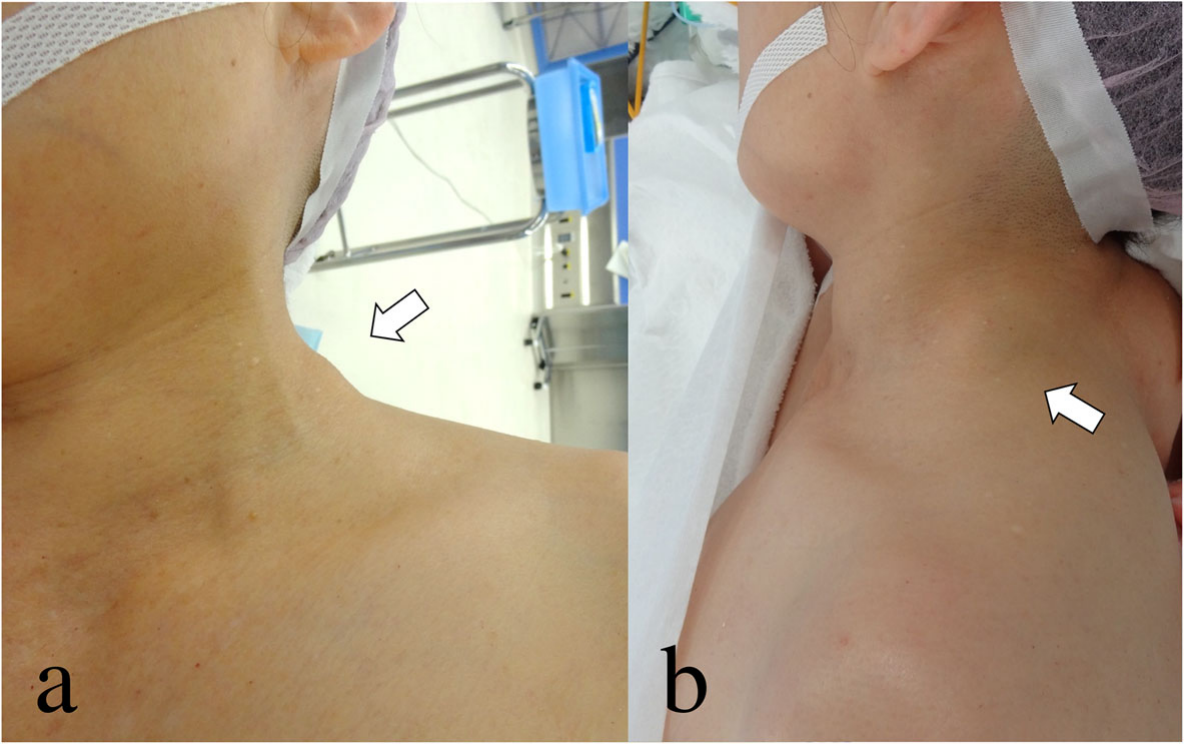
Clinical photographs of the patient. The tumor was located in the left levator scapulae muscle. **a** Front view. **b** Lateral view

**Fig. 2:**
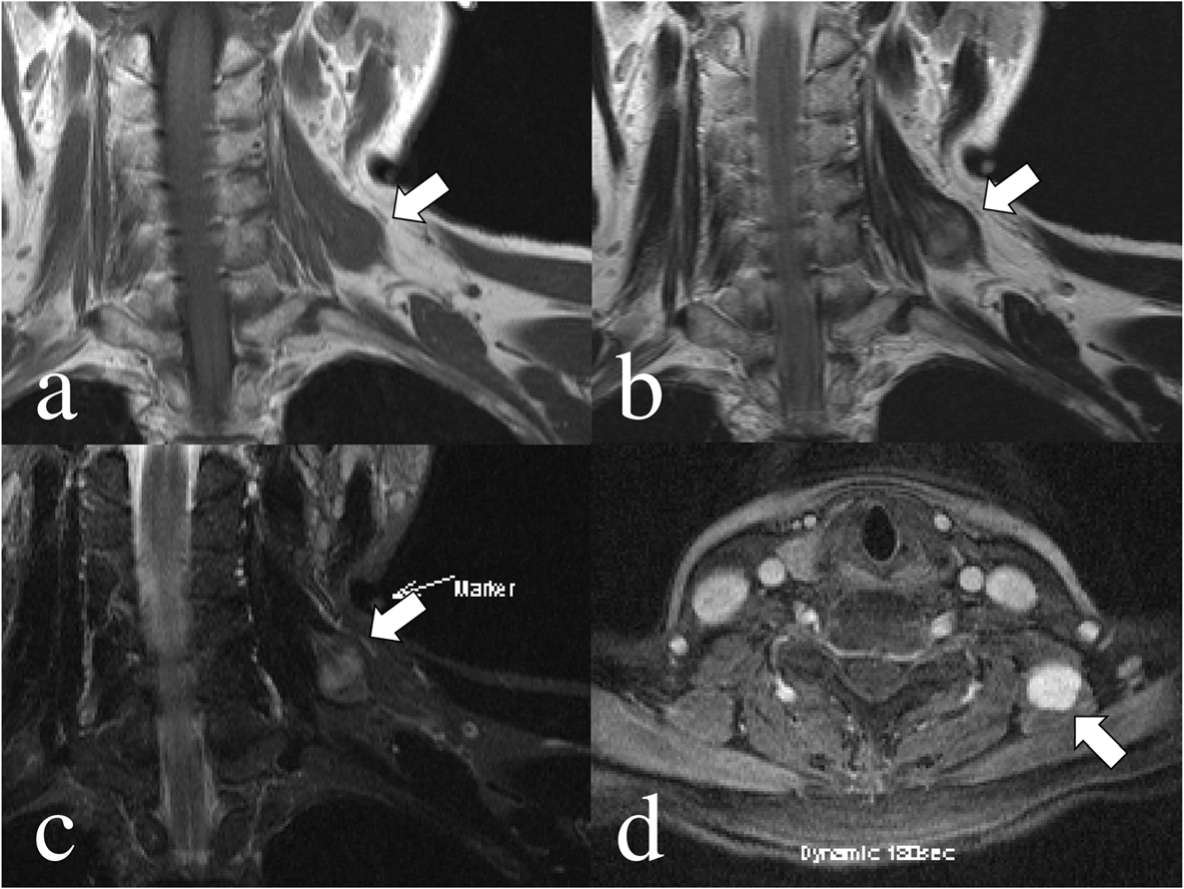
Preoperative magnetic resonance images (MRI) of the tumor. MRI revealed a tumor in the levator scapulae muscle. The mass with a size of 2.5 × 3.5 cm was well-demarcated and myxomatous (arrow). **a** T1 weighted image (WI). **b** T2WI. **c** short TI inversion recovery (STIR). **d** Contrast-MRI revealed good enhancement with gadolinium

**Fig. 3:**
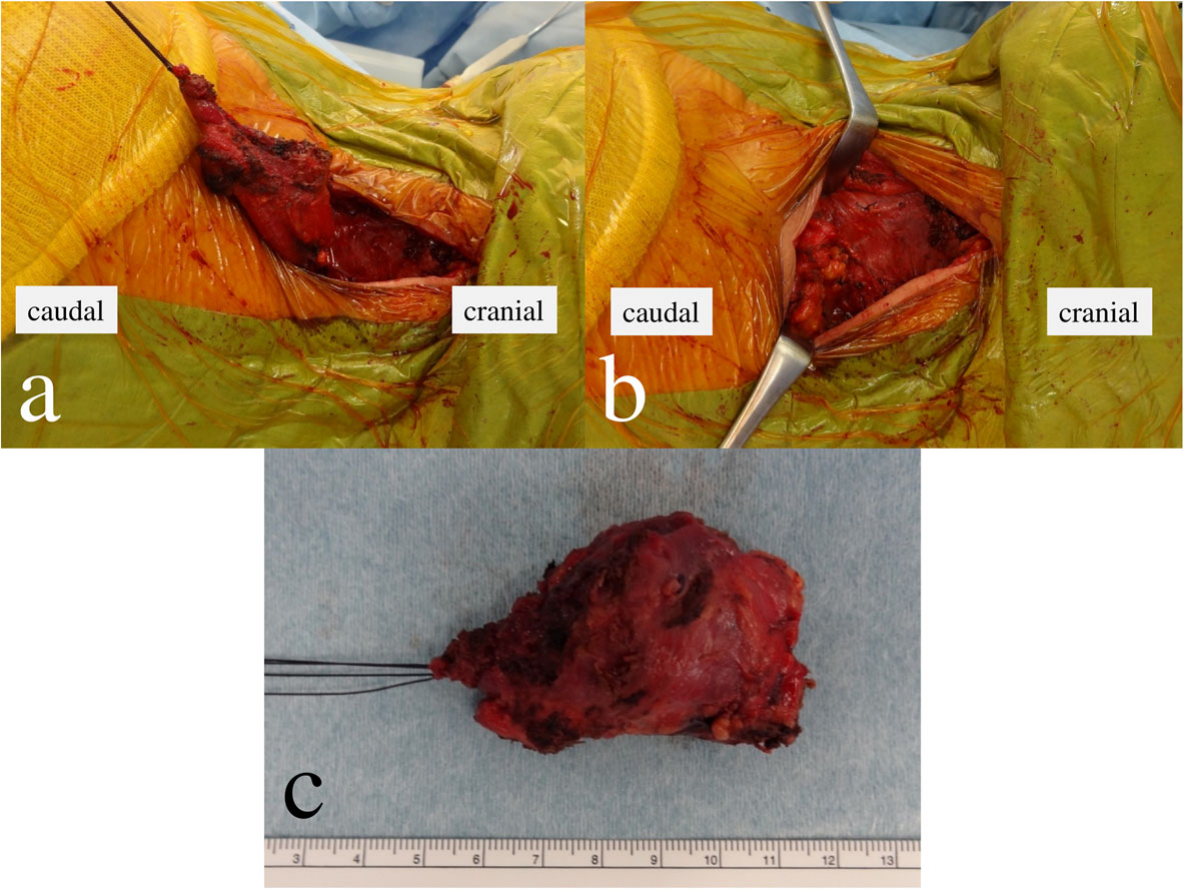
Clinical photograph of the surgery. The tumor was resected with a wide margin. **a** Intraoperative photograph showing the proximal side of the levator scapulae muscle was cut. **b** After wide excision. **c** Surgical specimen

**Fig. 4:**
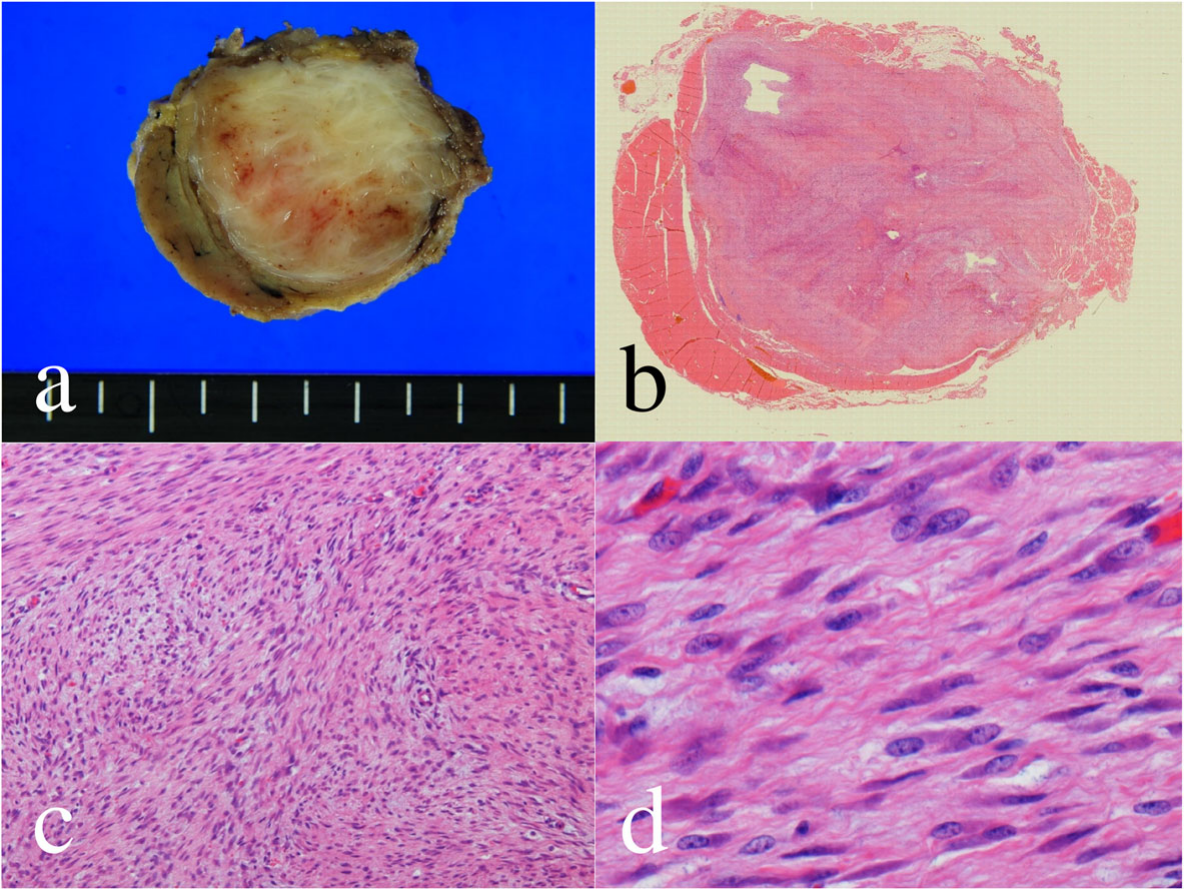
Histological findings of the tumor. **a** On sectioning, a well-demarcated nodular tumor, measuring 3.2 × 2.3 × 2.0 cm, was observed. The tumor was a solid, firm, gray-white mass, without necrotic foci or hemorrhage. **b** Magnification × 10. **c** Magnification × 100. **d** Magnification × 400. Histologically, the tumor was composed of spindle cells arranged in fascicles or storiform growth pattern in fibrous stroma. Neoplastic cells have ill-defined cytoplasm and nuclei with atypia. A few mitoses were observed, and there was not hypercellular area or necrosis

A 69-year-old female was admitted to our hospital because of a hard mass in her left neck six months prior (Fig. 1). On physical examination a palpable mass about the size of a quail egg was found to be located at the left neck. The mass had unclear boundaries, and was not tender. Magnetic resonance images (MRI) showed a soft tissue tumor of the left levator scapulae muscle with a size of 2.5 × 3.5 cm (Fig. 2). The mass appears isointense in T1-weighted image (WI) and heterogenously hyperintence in T2WI and short TI inversion recovery (STIR) image. Contrast-enhanced T1WI revealed marked enhancement with gadolinium. She had no medical, family, or surgical history. Needle biopsy specimens were obtained, and histological examination showed spindle cells with minimally atypia arranged with fascicular or storiform growth patterns in the fibrous stroma, suggestive of low-grade spindle cell sarcoma. Immunohistochemistry revealed focally positive for α-smooth muscle actin (αSMA), and negative for S-100. Based on the diagnosis of low-grade spindle cell sarcoma, tumor excision with a wide surgical margin was performed (Fig. 3). The cut surface of the surgically resected tumor was grayish-white (Fig. 4). The histological features comprised a proliferation of spindle-shaped tumor cells in the fibrous stroma with partially infiltrative growth into the surrounding muscle tissues. There was no pseudocapsule but the tumor boundary was nevertheless well-delineated. Inflammatory cells, atypia, and mitosis was rarely seen. Immunohistochemically, the tumor cells were positive for vimentin, αSMA (focally), and negative for HHF-35, desmin, H-caldesmon, CD34, and nuclear β-catenin. S-100 protein was focally positive. An ultrastructural analysis could not be performed because we had only paraffin-embedded material, unsuitable for electron microscope analysis. The lesion was diagnosed as LGMS.

The postoperative course was uneventful. The patient was alive with no evidence of local recurrence or metastasis eight years after the surgery without any additional therapy. She had no functional deficit and the shoulder range of motion was 150° in abduction, 150° in flexion, and 45° in extension at the final follow up. The patient had provided informed consent for publication of the case, and the study protocol was approved by Medical Ethics Committee of Kanazawa University.

## Discussion and conclusions

Literature reports published between 1998 and 2019 were identified using the search terms in PubMed and Google Scholar, excluding non-English language reports. We searched for synonyms of “low-grade myofibroblastic sarcoma” and “myofibroblastic sarcoma”. We checked reference lists of all included studies for additional sources. Our case was included in this review as well. A total of 103 cases from 48 reports were analyzed (Table 1) [1, 3, 4, 6–31, 33–51]. Age, location, symptoms, tumor size, infiltrative pattern, treatment method, local recurrence and outcomes were evaluated.

**Table 1:** Summary of the clinical features of previously reported 102 LGMS cases and the present case

Authors	Case	Age	Sex (M:F)	Size (cm)	Infiltrative pattern	EM	Treatment	FU (months)	LR	Oncological outcome
Mentzel et al. [1]	18	42 (19–72)	11:7	4.0 (1.4–17)	Yes (12) No (6)	Yes (4) No (14)	E (10) WE (2) E + RT (2) E + CTX (1) E + CRT (1) N/A (2)	35.9 (10–151)	Yes (3) No (8) N/A (7)	CDF (8) NED (2) AWD (1) N/A (7)
Montgomery et al. [3]	10	54.4 (28–73)	8:2	4.7 (1.5–12)	Mostly	Yes (6) No (4)	E (6) WE (1) E + RT (1) WE+RT (1) N/A (1)	48.1 (4–172)	Yes (4) No (5) N/A (1)	CDF (4) NED (2) N/A (4)
Meng et al. [29]	3	34 (14–74)	3:0	4.2 (3–5)	Yes (3)	Yes (1) No (2)	E + RT (3)	21.3 (16–27)	Yes (3)	NED (3)
Meng et al. [9]	14	30.5 (7–53)	9:5	4.6 (2.0–15.0)	N/A (14)	Yes (1) No (13)	E (8) E + CTX (4) E + RT (2)	28.3 (20–46)	Yes (5) No (8) N/A (1)	CDF (8) NED (5) N/A (1)
Cai et al. [23]	9	43.9 (6–73)	6:3	2.7 (1.5–5.0)	N/A (9)	No (9)	Scraped (1) E (3) E + RT (1) WE + RT (3) Lobectomy (1)	28.6 (11–44)	Yes (1) No (7) N/A (1)	CDF (7) NED (1) N/A (1)
Others [4, 6–8, 10–22, 24–28, 30, 31, 33–51]	48	43.8 (2–74)	23:25	4.1 (0.4–22)	Yes (28) No (2) N/A (18)	Yes (10) No (38)	E (18) E + RT (1) WE (21) WE+CTX (2) WE+RT (2) CRT (1) Others (2) N/A (1)	21.3 (6–72)	Yes (8) No (36) N/A (4)	CDF (36) NED (6) DOD (5) N/A (1)
Present case	1	69	female	3.5	Yes	No	WE	96	No	CDF

*LGMS* Low-grade myofibroblastic sarcoma, *M* Male, *F* Female, *N/A* Not applicable, *EM* Electron microscopy, *E* Excision, *WE* Wide excision, *CTX* Chemotherapy, *RT* Radiotherapy, *CRT* Chemo-radiotherapy, *LR* Local recurrence, *CDF* Continuous disease free, *NED* No evidence of disease, *AWD* Alive with disease, *DOD* Death of disease

Reported LGMS included 43 females and 60 males aged 2–75 years (median, 43). The number of patients aged < 18 years were 13 (12.6%), those aged 18 to 60 years were 67 (65.1%), and those aged ≥60 years were 23 (22.3%). The average tumor size was 4.4 cm (range: 0.4–22.0). As presented in Table 2, LGMS was located in the head and neck in 53 cases (51.5%); soft tissue 41 cases vs. bone 6 cases, trunk in 26 cases (25.2%); soft tissue 11 cases vs. bone 1 cases, and extremity in 24 cases (23.3%); soft tissue 12 cases vs. bone 7 cases. In the head and neck region, the most common site was the tongue [1, 6, 8–10, 13, 14], followed by the larynx [17–20, 47], and gingiva [3, 12, 15, 16]. LGMS was also observed in the mandible [10, 23], face [23], skull [9], acoustic meatus [9], and deep tissue spaces, including the parapharyngeal space [28] as well as throughout the head and neck region. Some authors have reported LGMS of bone [3, 9, 23, 36, 42, 43, 45]. LGMS of the bone was a total of 14 cases (13.6%) and were located in the femur in five cases (4.9%), mandible in three cases (2.9%), maxilla in two cases (1.9%), tibia in two cases (1.9%), hard palate in one case (1.0%), and sacrum in one case (1.0%). In this series, the most common site of LGMS in the extremities was the femur, followed by groin in four cases, and thigh in three cases. There was another large case series reported by Chan et al. [52] They reported 49 cases of LGMS in the USA, and the most common sites were the extremities in 40.8% of cases, followed by the head and neck region with 26.5% of cases [52]. In contrast to their study, these 103 cases revealed that LGMS was commonly located in the head and neck, especially in the oral cavity.

**Table 2:** Comparisons of clinical features according to location of previously reported 102 LGMS cases and the present case

	Head and neck	Trunk	Extremity	Total
Number of cases	53 (51.5%)	26 (25.2%)	24 (23.3%)	103
Size (cm)	2.7 cm (0.4–5.9)	7.5 cm (1.2–22.0)	4.9 cm (1.5–11.0)	4.4 cm (0.4–22.0)
LGMS of soft tissue	41/47 (87.2%)	11/12 (91.6%)	12/19 (63.3%)	64/78 (82.0%)
SC (+D)	7/47 (14.9%)	4/12 (33.3%)	4/19 (21.1%)	15/78 (19.2%)
SM (+Mu)	21/47 (44.7%)	1/12 (8.4%)	0/19 (0%)	22/78 (28.2%)
IM	11/47 (23.4%)	2/12 (16.6%)	4/19 (21.1%)	17/78 (21.8%)
Others	2/47 (4.2%)	4/12 (33.3%)	4/19 (21.1%)	10/78 (12.8%)
LGMS of bone	6/47 (12.8%)	1/12 (8.4%)	7/19 (36.7%)	14/78 (18.0%)
Infiltrative pattern				
Yes	28/29 (96.6%)	10/14 (71.4%)	6/9 (66.7%)	44/52 (84.6%)
No	1/29 (3.4%)	4/14 (28.6%)	3/9 (33.3%)	8/52 (15.4%)

*LGMS* Low-grade myofibroblastic sarcoma, *D* Dermis, *SC* Subcutaneous, *Mu* Mucosa, *SM* Submucosa, *IM* Intramuscular

In most cases, patients present with a painless swelling or an enlarged mass, and pain or related symptoms are rarely reported. In these 103 cases, 33 out of 50 patients had swelling, and 16 out of 50 patients had pain. Chan et al. [52] reported non-head and neck LGMS with a significantly higher number of cases with tumor size > 4 cm. They reported that the mean tumor size in the head and neck region was 3.2 cm (range, 1.0–7.7); in the non-head and neck region tumors, the mean size was 7.1 cm (range, 2.4–24.0). In these 103 cases, the tumor size located in head and neck, trunk, and extremity averaged 2.7 cm (range, 0.4–5.9), 7.5 cm (range, 1.2–22.0), and 4.9 cm (range, 1.5–11.0), respectively. LGMS in the head and neck region was smaller than in other regions. The levator scapulae muscle is located at a relatively superficial position [53]; therefore, the present case had the tumor located at a palpable depth, even though it was relatively small.

MRI revealed that T1weighted image (WI) signal was mostly an equal signal, T2WI signal was an iso-to-high signal [22, 37, 41, 44]. Gadolinium enhancement T1WI showed enhancement of the tumor [22, 41]. The border of the tumor on the MRI was varied; some author reported well demarcated tumor [7, 13, 44], but the others were ill-defined [10, 41, 42]. Morii et al. [41] and Niu et al. [50] reported the usefulness of ^18^F-Fluorodeoxyglucose-positron emission tomography (FDG-PET)/ computed tomography (CT) for diagnosing LGMS. They reported abnormally increased FDG metabolism and the maximum standard uptake value (SUV max) of the tumor. In previous reports, SUVmax of LGMS were 2.8–9.8 [41, 50]. They suggested that the high capacity of glucose utilization is a possible reflection of LGMS. Differential diagnoses for this tumor include leiomyosarcoma, low-grade fibrosarcoma, well-differentiated osteosarcoma, desmoplastic fibroma, inflammatory myofibroblastic tumor, nodular fasciitis, and fibromatosis. Leiomyosarcoma is a tumor that needs to be most carefully differentiated among them [3, 4, 6, 38, 41, 48]. In these 103 cases, the preoperative histologic assessment was performed in 21 patients. Thirteen out of 21 patients had a record of the details (fine needle aspiration cytology; FNAC, core needle biopsy; CNB, or open incisional biopsy; OIB). A FNAC was performed in three lesions, a CNB was performed in six lesions, and an OIB was performed for six lesions. Both the CNB and OIB were performed in two lesions. Two out of six lesions evaluated by CNB were diagnosed as benign lesions (benign fibrohistiocytoma and myofibroblastoma). The other four lesions were diagnosed as malignant lesions (atypical spindle cell with numerous mitotic figures, synovial sarcoma, malignant mesenchymal tumor, and low grade spindle cell sarcoma) However, the lesions evaluated by OIB were diagnosed as LGMS in three cases, myofibroblastic sarcoma in one case, low grade fibrosarcoma in one case, and low grade sarcoma in one case. There were no malignant cells in three lesions evaluated by FNAC. The positive margin ratios in the biopsy group (12 cases) and non-biopsy group (26 cases) were 16.7 and 38.4%, respectively. Two out of 12 patients in biopsy group was positive surgical margin. Both cases had infiltrative growth pattern and the tumor locations were deep (larynx and left maxillary sinus) [18, 47]. Regarding to the LGMS in the upper aerodigestive tract, Meng et al. [29] reported that owing to the diverse histologic appearance in the same tumor of myofibroblastic sarcoma; misdiagnosis may occur in small and superficial biopsy samples. Montebugnoli et al. [12] also reported that an OIB must be performed, reaching an adequate submucosal depth because a misinterpretation can result from the specimen being sampled from the tumor surface, which contains mainly the granulation tissue-like and hypocellular areas but not the atypia hypercellular area. However, if the tumor location is trunk or extremity, CNB are sometimes preferable because of its convenience, which can be performed as day surgery under local anesthesia at the outpatient clinic [54]. In the present case, the histological grade was accurate by CNB, and wide excision was performed. If the tumor is not sampled by CNB, OIB is recommended to prevent inadequate excision. If the tumor size is less than 3 cm, an excisional biopsy can be indicated [55].

Recently, LGMS has been defined as a distinct entity under a new classification of soft tissue tumors [2]. In the 2002 WHO classification of soft tissue and bone tumor pathology and genetics, LGMS was for the first time classified as a distinct entity. In the subsequent versions, it is still referred to as LGMS and classified as part of the fibroblastic/myofibroblastic tumor category. LGMS is classified as an intermediate (rarely metastasizing) type of myofibroblastic tumor [2]. Myofibroblasts have been characterized as mesenchymal spindle cells that share features of both fibroblasts and smooth muscle cells. Some authors consider that electron microscopy is the gold standard for diagnosis of the presence of myofibroblasts. However, only 22 (21.4%) of 103 cases were reviewed by electron microscopy in this series. Ultrastructurally, myofibroblasts are characterized by myofilaments with focal density (stress fiber) and prominent rough endoplasmic reticulum (RER) [56–58]. In contrast, it is controversial whether fibronexus, occasionally observed in reactive myofibroblasts and myofibroblastic tumors, is a specific and essential feature of myofibroblasts [56–59]. Some authors reported that recognizing purely myofibroblastic differentiation is difficult without electron microscopic examination, but agree that neoplastic cells have poorly developed ultrastructural features that may not identifiable in all cases [4, 60]. Although electron microscopic examinations were not available in the present case, the extensive vimentin- and actin-positive and h-caldesmon-negative immunohistochemical staining, and their eosinophilic wavy cellular features, supports their myofibroblastic differentiation.

Surgery is the primary treatment modality for LGMS [6, 10, 32, 39]. Adjuvant therapies such as chemotherapy and radiotherapy have also been used in some cases [1, 3, 29, 33, 61], although the optimal treatment of LGMS remains ill-defined. Peng et al. [33] reported no recurrence five years after surgery and adjuvant chemotherapy for LGMS of the pancreas. However, Humphries et al. [43] reported that chemotherapy does not seem to be effective. About radiotherapy, Khosla et al. [18] reported that the patient underwent postoperative radiotherapy because of margin involvement, and the patient was alive and disease-free 14 months after surgery. Because LGMS is extremely rare, the standardization of its treatments, including surgery, chemotherapy, and radiotherapy, requires further investigation. Chan et al. [52] reported a 5-year overall survival of 71.6% and disease-specific survival of 76.3%.

LGMS usually affects deep soft tissue sites and is often poorly circumscribed with fascicles and individual cells infiltrating between muscle fibers, although focal circumscription is not unusual [4]. Forty-four (84.6%) out of 52 cases had infiltrative growth patterns in these 103 cases. Therefore, local LGMS recurrences are common, whereas metastasis occurs only rarely [1, 3, 52] and then only after a prolonged period. Regarding the prognosis of LGMS, Montgomery et al. [3] reported that four of nine LGMSs recurred. The median duration to recurrence was 11.5 months. Yamada et al. [30] reported a recurrence rate of approximately 38%, which correlated with the tumor size. In these 103 cases, 24 out of 90 cases (26.7%) had a local recurrence (Table 3).

**Table 3:** Comparisons of oncological outcomes according to location of previously reported 102 LGMS cases and the present case

	Head and neck	Trunk	Extremity	Total
Oncological outcome				
CDF	35/48 (72.9%)	15/23 (65.2%)	14/18 (77.8%)	64/89 (71.9%)
NED	11/48 (22.9%)	5/23 (21.7%)	3/18 (16.7%)	19/89 (21.4%)
AWD	0/48 (0%)	1/23 (4.4%)	0/18 (0%)	1/89 (1.1%)
DOD	2/48 (4.2%)	2/23 (8.7%)	1/18 (5.5%)	5/89 (5.6%)
Local recurrence				
Yes	13/49 (26.5%)	6/22 (27.3%)	5/19 (26.3%)	24/90 (26.7%)
No	36/49 (73.5%)	16/22 (72.7%)	14/19 (73.7%)	66/90 (73.3%)
Distant metastasis				
Yes	1/49 (2.0%)	2/23 (8.7%)	1/19 (5.3%)	4/91 (4.4%)
No	48/49 (98.0%)	21/23 (91.3%)	18/19 (94.7%)	87/91 (95.6%)

*LGMS* Low-grade myofibroblastic sarcoma, *CDF* Continuous disease free, *NED* No evidence of disease, *AWD* Alive with disease, *DOD* Death of disease

In conclusion, LGMS is still an uncommon malignant tumor, occurring mostly in those 18 to 60 years old with a male preponderance. LGMS occurs most commonly in the head and neck region, followed by the trunk and extremities. The tumors’ size in the trunk is larger than in other sites. An infiltrative pattern was detected in more than 80% of cases. Local recurrence was 26.7%, and distant metastasis was 4.4%. Therefore, it is important to diagnose LGMS with biopsy and to excise the tumor with an adequately wide margin. To the best of our knowledge, this is the first case report of LGMS arising in the levator scapulae muscle. The tumor was widely resected, and no recurrence was observed over eight years.

## Data Availability

All data generated or analyzed during this study are included in this article.

## Abbreviations

LGMS: Low-grade myofibroblastic sarcoma
MRI: Magnetic resonance images
α-SMA: α-smooth muscle actin
FDG-PET: Fluorodeoxyglucose-positron emission tomography
CT: Computed tomography
